# Investigation of biofilm formation and the associated genes in multidrug-resistant *Salmonella pullorum* in China (2018–2022)

**DOI:** 10.3389/fvets.2023.1248584

**Published:** 2023-08-31

**Authors:** Wenyan Chen, Ziheng Xu, Changcheng Li, Can Wang, Min Wang, Jingzhen Liang, Ping Wei

**Affiliations:** ^1^Institute for Poultry Science and Health, Guangxi University, Nanning, China; ^2^School of Public Health and Management, Guangxi University of Chinese Medicine, Nanning, China

**Keywords:** *Salmonella pullorum*, biofilm, multidrug resistance, minimal biofilm eradication concentration, optimal biofilm formation condition

## Abstract

The study explored the biofilm (BF) formation capacity, BF-related gene profiles, and the trends in antimicrobial resistance (AMR) of *Salmonella pullorum* (SP) strains over several years. A total of 627 SP strains were isolated from 4,540 samples collected from chicken farms in Guangxi, China during 2018–2022. The BF-forming capacity of these isolates was assessed using crystal violet staining, and the presence of eight BF-related genes (*csgA, csgB, csgD, ompR, bapA, pfs, luxS*, and *rpoS*) in BF formation-positive strains was determined through Polymerase Chain Reaction (PCR) analysis. Antimicrobial susceptibility test was conducted to investigate the AMR of the isolates. Minimum Inhibitory Concentration (MIC) and Minimal Biofilm Eradication Concentration (MBEC) of nine SP-BF strains were determined using the broth microdilution method to assess the impact of BF formation on AMR. Additionally, the Optimal Biofilm Formation Conditions (OBFC) were investigated. The results indicated that 36.8% (231/627) of the strains exhibited a positive BF-formation capacity. Among these, 24.7% (57/231) were strong BF producers, 23.4% (54/231) were moderate BF producers, and 51.9% (120/231) were weak BF producers. Analysis of the eight BF-related genes in SP-BF strains revealed that over 90% of them were positive for all the genes. Antimicrobial susceptibility test conducted on the isolates showed that 100% (231/231) of them exhibited resistance to at least one antibiotic, with 98.3% (227/231) demonstrating multidrug resistance (MDR). Both MIC and MBEC measurements indicated varying degrees of increased AMR after BF formation of the bacteria. The optimal conditions for BF formation were observed at 37°C after 48 h of incubation, with an initial bacterial concentration of 1.2 × 10^6^ CFU/mL. Notably, NaCl had a significant inhibitory effect on BF formation, while glucose and Trypticase Soy Broth (TSB) positively influenced BF formation. The results of the study emphasized the need for effective preventive and control strategies to address the challenges posed by the BF formation and MDR of SP in the field.

## 1. Introduction

*Salmonella pullorum* (SP) is a globally distributed chicken-specific pathogen that causes acute systemic and chronic localized diseases in poultry, known as pullorum disease (PD), which negatively impacts hatching rates and impairs the growth performance of surviving chicks (1). Transmission of SP can occur vertically through breeding eggs or horizontally through contaminated environments. An important characteristic of SP is its capacity to form a biofilm (BF) in the external environment, which poses challenges for clinical prevention and control (2). BF consists of bacterial cells and extracellular polymeric substances (EPSs). The EPS matrix comprises extracellular polysaccharides, proteins, lipids, nucleic acids (including extracellular DNA and RNA), and other biomolecules that are secreted by bacteria (3). Bacteria within the BF are shielded by the EPS, which provides protection against adverse environmental pressure and confers increased resistance to antibiotics compared with the planktonic bacteria.

The formation of BF is a dynamic and cyclic process that occurs in a progressive manner. The four developmental steps of BF are discernable as reversible attachment, irreversible attachment, maturation, and dispersion (4). The formation of BF is influenced by the interplay of environmental conditions, cell density, and signaling molecules, with the quorum sensing (QS) system playing a crucial role in this process. By activating specific signaling molecules, the QS system coordinates the expression of various genes related to BF formation, including those encoding extracellular proteins, sigma factors, and curli fibers. It also governs the entire process of BF establishment, maintenance, stress resistance, and dispersal (5). With an elaborate three-dimensional structure, mature BFs establish an interconnected network that facilitates the transport of water, nutrients, and metabolites. Moreover, planktonic bacteria continuously release into the external environment. These released bacteria have the potential to form BF in different sites or opportunistically infect susceptible hosts under favorable conditions, thereby contributing to the persistence of chronic infections over an extended period.

The control of PD primarily involves eradication strategies and employs antibiotic treatment (6). However, the effectiveness of antibiotics in treating PD is limited and, conversely, partly increases the susceptibility of chickens to *Salmonella*. This is attributed to the disturbance of the microbial balance caused by the inhibitory effects of antibiotics on the normal gut microflora (7). As the prolonged presence of infection on farms results in a substantial increase in antimicrobial consumption, the antimicrobial resistance (AMR) and even the multidrug resistance (MDR, non-susceptibility to at least one agent in three or more antimicrobial categories) of SP have been gradually increasing over the years (8, 9). Moreover, once SP forms a BF, its physical resistance and sensitivity to antimicrobial factors decrease, making it more challenging to eradicate antibiotics. Furthermore, the AMR-BF serves as natural reservoirs for antimicrobial resistance genes (ARGs), facilitated by the dense populations and close cellular proximity of EPS, increasing the risk of ARG transmission among bacterial populations and even to potential zoonotic pathogens (10). Therefore, investigating the MDR characteristics and BF formation capacity, the underlying mechanisms of SP hold immense significance in devising effective preventive and control measures.

## 2. Materials and methods

### 2.1. Sample collection

All studies were conducted on commercial chickens, and the company performing the study followed ethical standards for commercial chicken production. The live birds used for the tissue collection were killed by carbon dioxide narcosis. A total of 4,540 samples were collected from six large-scale breeding chicken farms in Guangxi, China, during 2018–2022, covering the entire production chain of breeders. The samples encompassed diverse sources, including liver and cecal samples from deceased embryos, failed hatchling embryos, and culled 1-day-old chicks (each sample consisted of 30–50 specimens per farm, with a total of 2,250 specimens). Additionally, various tissues including the heart, liver, spleen, lung, ovary, and oviduct were obtained from a random selection of inspection breeders (15 breeders per farm, with a total of 250 breeders). Moreover, cotton swabs were used to collect samples from various surfaces in the hatcheries, including incubators, conveyor belts, hatching eggs, chick trays, and the meconium (each sample consisted of 10–30 specimens per farm, with a total of 2,040 specimens). The number of specimens collected per sample varied depending on the degree of SP eradication and the specific environmental conditions in each farm. The sampling frequency for each farm averaged once per year. Chicken tissue samples were collected under sterile conditions, while swab samples were collected directly on-site and placed in the Eppendorf tube with 1 ml of buffered peptone water (BPW) (Huankai, Guangdong, China).

### 2.2. *Salmonella* isolation and identification

*Salmonella* isolation and identification were conducted following the China agricultural industry standard methods for diagnostic techniques for avian paratyphoid and pullorum disease (11). In brief, 1 g of minced tissues or swab samples in 1 ml of BPW was placed in 9 ml BPW for pre-enrichment culture at 37°C for 24 h. Overall, 1 ml of the pre-enrichment cultures was transferred into 9 ml of Selenite Cystine Broth (SC) (Huankai, Guangdong, China) and incubated at 37°C for 24 h. Subsequently, SC culture was streaked onto xylose lysine desoxycholate (XLD) agar (Huankai, Guangdong, China) and stabbed into triple sugar iron (TSI) agar slants (Huankai, Guangdong, China), and then incubated at 37°C for 24 h. Presumptive *Salmonella* colonies were subjected to biochemical tests using bacterial microbiochemical reaction test tubes (Hangzhou Microbiology, Zhejiang, China) for further confirmation, including ornithine de-carboxylation test, dulcitol fermentation test, and gas production from glucose. Moreover, the specific genes *invA* and *rfbs* used for SP identification were detected using polymerase chain reaction (PCR). The serotypes of all isolated strains were determined using the slide agglutination test and the Kauffman–White serotyping method (12), with antisera against the poly A-F, 9, and 12 of O-type antigens of *Salmonella* (Tianrun, Ningbo, China).

### 2.3. BF formation capacity determination

BF formation capacity was assessed using crystal violet staining on 627 strains of SP isolates, following the methodology by Agarwal et al. (13), with some modifications. In brief, bacterial suspensions at the logarithmic growth phase (20 μl) were added to each well of a 96-well plate containing 180 μl of Trypticase Soy Broth (TSB). The plate was then incubated at 37°C for 48 h. After removing the culture medium, the wells were washed gently three times with phosphate-buffered saline (PBS) (Huankai, Guangdong, China) to eliminate non-adherent cells. Subsequently, the BF was fixed with 200 μl of methanol (Ourchem, Shanghai, China) for 15 min and stained with 200 μl of 1% crystal violet (Solarbio, Beijing, China) for 10 min in the absence of light. The excess stain was gently washed away with PBS, and 200 μl of anhydrous ethanol (Macklin, Shanghai, China) was added to each well to dissolve the bound stain. Each strain was tested in triplicate, and a blank control was included. The absorbance at 595 nm was measured as the optical density (*OD*) value. Following the criteria described by Stepanović et al. (14), the average *OD* value of the triplicates for each strain was compared with the *OD* value of the blank control (*ODc*). Based on this comparison, the strains were categorized into four groups according to their BF-forming capacity: strong BF producers (*OD* > 4 *ODc*), moderate BF producers (2 *ODc* < *OD* ≤ 4 *ODc*), weak BF producers (*ODc* < *OD* ≤ 2 *ODc*), and non-BF producers (*OD* ≤ *ODc*).

### 2.4. Detection of BF-related genes

The detection of eight BF-related genes, namely, *csgA, csgB, csgD, ompR, bapA, pfs, luxS*, and *rpoS*, was performed using PCR. The total DNA of the strains was extracted using the boiling method (15). The primers were designed using *Oligo* 7 and *Primer* 5 software and synthesized by Huada Gene Company (Beijing, China). The primer sequences and optimal annealing temperatures for PCR amplification are presented in Table 1. Positive PCR products were subsequently sent to Huada Gene Company for sequencing.

**Table 1:** Primer sequences, target genes, amplicon sizes, and annealing temperature are used in PCR.

**Gene categories**	**Target gene**	**Primers sequences (5^′^-3^′^)**	**Amplified segment (bp)**	**Annealing temperature**
*Salmonella pullorum*	*invA*	CTATTTTAAATTCCGTGAAGCAA	378	55°C
		ACTTCATCGGAATAATTTACCAC		
	*rfbs*	AGAAAGCAATATTCTTATGCCTA	385	51°C
		TCAATACTATGAAATTTGGGGAA		
Curli	*csgA*	TGCCCGTAAATCTGAAACGACCA	251	57°C
		CGTTGTTGCCAAAACCAACCTG		
	*csgB*	GACAAATTATGATCTGGCTCGTT	282	54°C
		ATAGCCGCACTATTACCGTA		
	*csgD*	GTGTTTTACGCTACTGAAGACCA	253	54°C
		GATGTGTCTTAACCGTATTCTCG		
Protein	*ompR*	ATTTAGCCTGAAATTCATACCAG	179	53°C
		TGCTCGGTCAGATAACGTTC		
	*bapA*	TTAACTATGTCAACAACGGTCCT	342	57°C
		TATTCAGCACAAACAGGTACTCG		
Quorum sensing	*pfs*	TTACCACGACGCTGATGTGACC	303	60°C
		TCAGAAATAGCGCGAACCACCAC		
	*luxS*	CATTGCCCGTCATATTCTGGA	296	53°C
		TGTGATCAATACACTCTGGCATC		
Sigma factor	*rpoS*	GGCGATCATGAACCAAACCCGTA	416	59°C
		TTTCACGGCCTACATCTTCCAGT		

### 2.5. Antimicrobial susceptibility test of the BF formation-positive SP strains

The Kirby–Bauer disk diffusion method was used for the AST (16). *Escherichia coli* ATCC25922 was used as the control strain. Susceptibility breakpoints were derived from the Clinical Laboratory Standards Institute (17). Moreover, the breakpoints for amoxicillin and florfenicol were referenced by Xu et al. (18). A total of 17 antimicrobial agents were applied (Hangzhou Microbiology, Zhejiang, China), such as ampicillin (AMP, 10 μg), amoxicillin (AMC, 20 μg), cefotaxime (CTX, 30 μg), ceftazidime (CAZ, 30 μg), ceftriaxone (CRO, 30 μg), trimethoprim/sulfamethoxazole (SXT, 1.25/23.75 μg), methoxypyrimidine (TMP, 5 μg), sulfisoxazole (SIZ, 300 μg), nalidixic acid (NAL, 30 μg), ciprofloxacin (CIP, 5 μg), amikacin (AKN, 30 μg), streptomycin (STP, 10 μg), kanamycin (KAN, 30 μg), gentamicin (GEN, 10 μg), nitrofurantoin (NIT, 300 μg), tetracycline (TET, 30 μg), and florfenicol (FFC, 30 μg).

### 2.6. Determination of minimum inhibitory concentration and minimal biofilm eradication concentration

MIC was determined by the broth microdilution method (19). The MIC values of CAZ, CIP, GEN, NIT, and TET were determined for nine strains with varying degrees of BF-forming capacity. In brief, the bacterial suspensions were prepared in an inoculum of 0.5 McFarland (McF). The concentrations of antibiotic solutions ranged from 2,048 μg/ml to 1 μg/ml, which were obtained from a series of 2-fold dilutions. The bacterial suspension and antibiotic solution were mixed in the culture plates and incubated at 37°C for 20 h. MBEC was defined as the lowest concentration of antibiotic required to eliminate the BF. The methodology described by Cruz et al. (20) was utilized for this purpose. The BF strains were pre-cultured in 200 μl TSB in the 96-well plates at 37°C for 48 h, and the subsequent steps were performed following the same procedure as described earlier. Each experiment was replicated three times.

### 2.7. Optimal biofilm formation conditions and nutritional factors of SP strains

The OBFC of SP was investigated through single-factor experiments. Various factors were examined, including the initial bacterial concentrations (ranging from 1.5 × 10^8^ to 1.5 × 10^1^ CFU/ml), culture times (0 h, 3 h, 6 h, 9 h, 12 h, 24 h, 36 h, 48 h, and 60 h), temperatures (25°C, 30°C, 35°C, 37°C, 40°C, 45°C), and varying concentrations of TSB medium, glucose, and NaCl (21). The formation of BF was then quantified using the crystal violet staining method as described earlier.

## 3. Results

### 3.1. BF formation capacity of SP isolates from large-scale breeding farms

**Figure 1:**
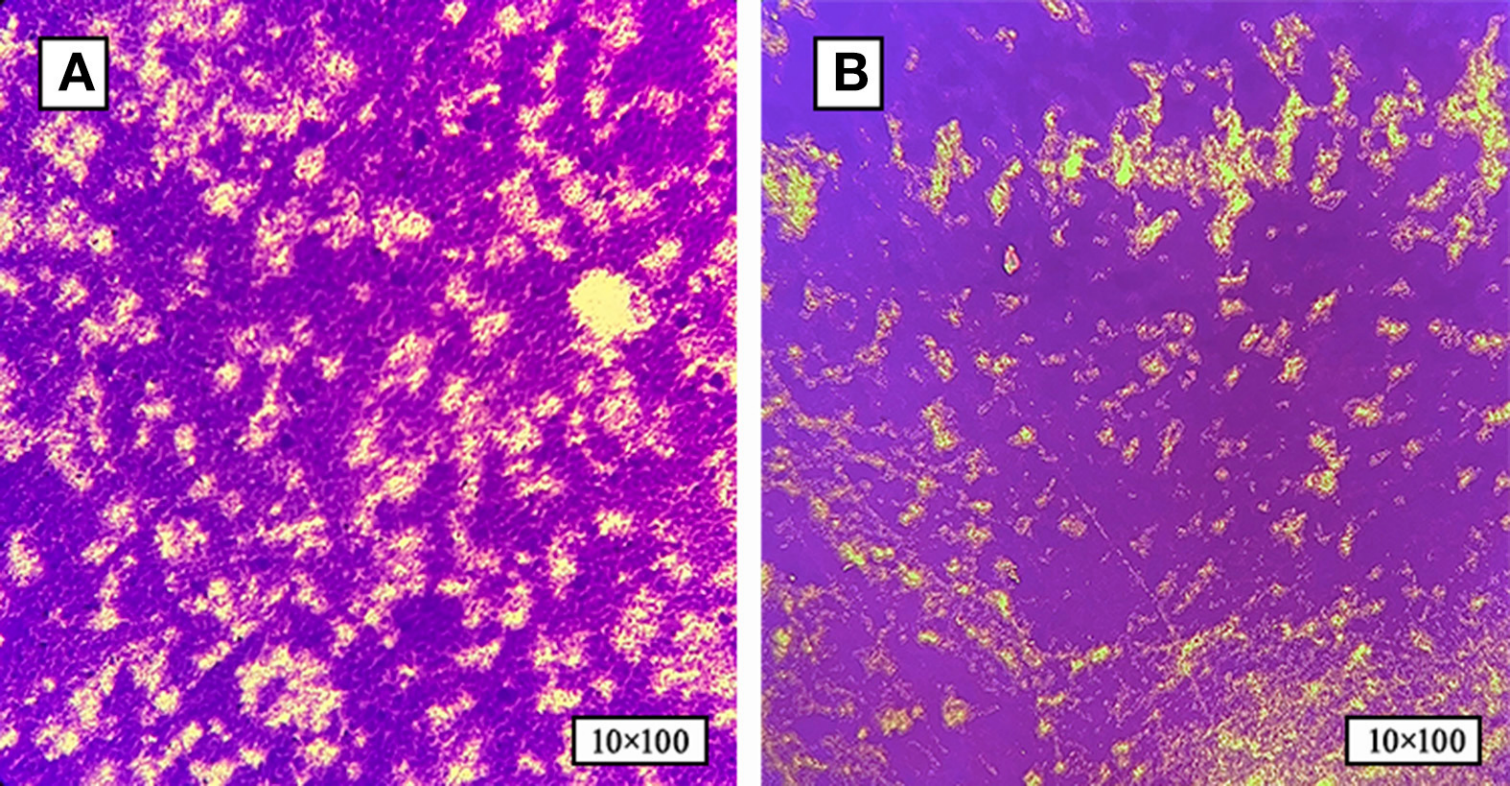
The formation of SP-BF was assessed by crystal violet staining at various time intervals. **(A)** Biofilm formation of SP at 24 h post-inoculation time point. **(B)** Biofilm formation of SP at 48 h post-inoculation time point. SP-BF gradually increases over time intervals.

**Figure 2:**
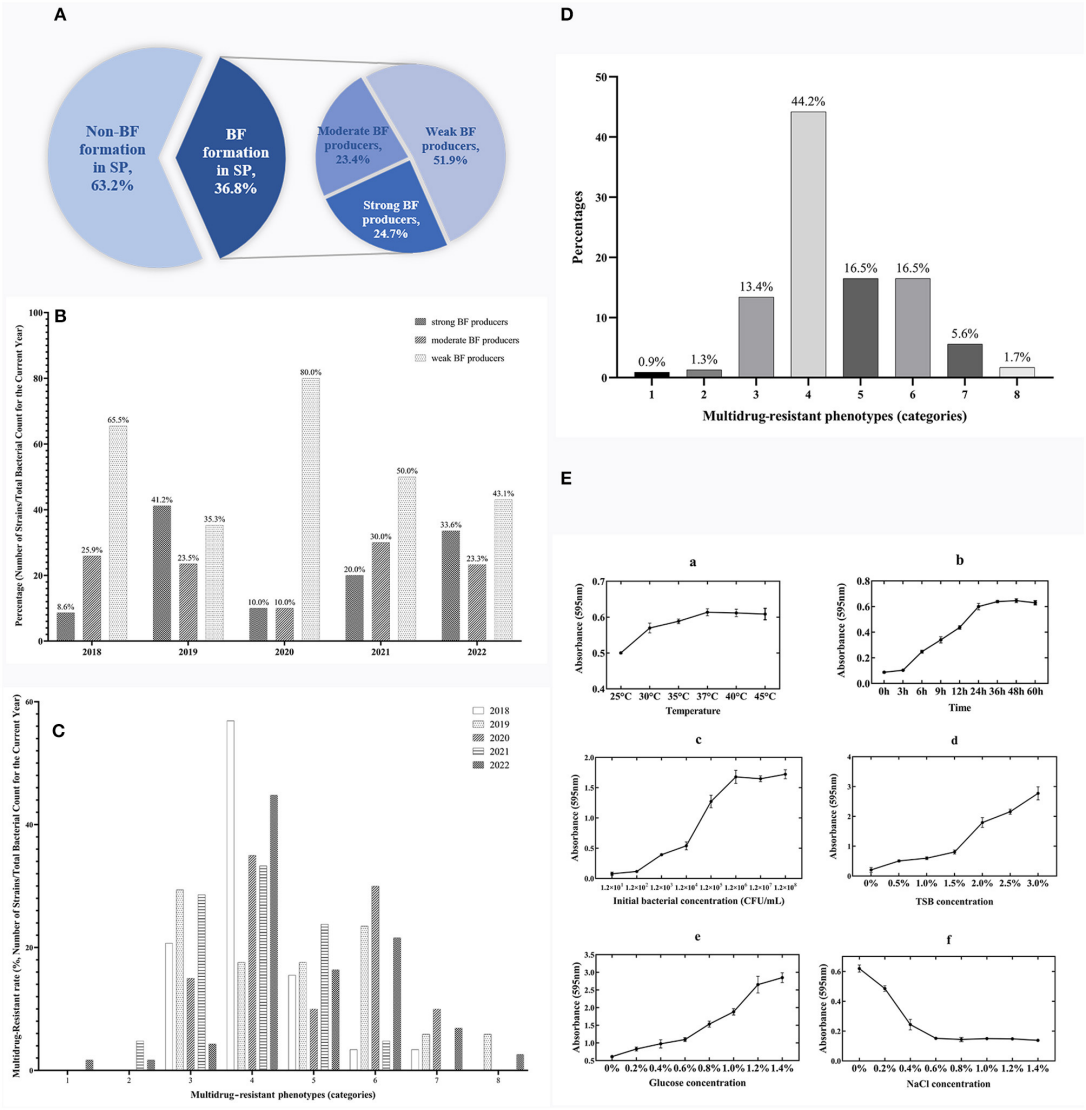
Temporal surveillance of BF-forming capacity, proportions, and MDR phenotypes in BF formation-positive SP isolates (2018–2022): Investigating the OBFC and nutritional influences for SP-BF. **(A)** Proportions of strong, moderate, and weak BF producers among 231 BF formation-positive strains from 627 total SP isolates. **(B)** BF-forming capacity of SP from 2018 to 2022. **(C)** Temporal surveillance of MDR in SP-BF from 2018 to 2022. **(D)** MDR phenotype of BF formation-positive SP. **(E)** The OBFC and nutritional factors for SP-BF. The subfigures (a–f) represent the effects of different factors on BF formation, specifically temperature, time, initial bacterial concentration, TSB concentration, glucose concentration, and NaCl concentration.

Based on the bacterial morphology, biochemical characteristics, serotyping test, and PCR detection results, 627 strains of SP were isolated and identified from a substantial cohort of 4,540 field samples from six large-scale breeding farms during 2018–2022. Subsequently, the BF formation capacities of all the SP strains were evaluated. The morphological characteristics of BF at different time intervals are depicted in Figure 1, as observed through crystal violet staining. The results showed that 36.8% (231/627) of the strains exhibited BF formation capacity. Among the BF formation-positive strains, 24.7% (57/231) were strong producers, 23.4% (54/231) were moderate producers, and 51.9% (120/231) were weak producers, as shown in Figure 2A. Notably, during the years 2020–2022, an increased proportion of SP strains with moderate and strong BF formation capacity was found. In 2020, it was 10% for both categories, while in 2021, it rose to 30% for BF moderate producers and 20% for BF strong producers. By 2022, the percentages reached 23% and 34%, respectively. The specific data are presented in Figure 2B.

### 3.2. Detection of the BF-related genes in BF formation-positive strains of SP

**Figure 3:**
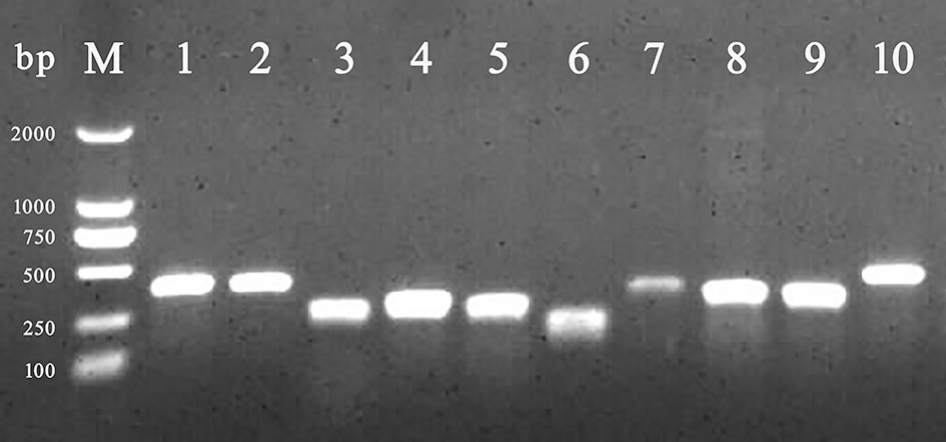
PCR amplification of BF-related genes. M: DL 2,000 DNA marker; from lanes 1 to 10 were the detection results of *invA, rfbs, csgA, csgB, csgD, ompR, bapA, pfs, luxS*, and *rpoS* gene, respectively.

Among BF formation-positive strains, the detection rates of the eight BF-related genes were ranked from high to low as follows: protein, sigma factor, curli fibers, and QS system. The detailed findings are provided in Table 2, and the PCR results are illustrated in Figure 3. All BF-related genes, except for *csgB, csgD*, and *pfs* genes, were detected in every strain tested (100%, 231/231). Over 94% of the tested strains were positive for curli fiber-related genes (*csgA, csgB*, and *csgD*). Moreover, the detection rate of the *pfs* gene was 90.5% (209/231).

**Table 2:** Detection rates of BF-related genes in the BF formation-positive SP strains.

**Gene categories**	**Target gene**	**Detection rate (no. of positive/total)**
Curli fiber	*csgA*	100% (231/231)
	*csgB*	94.8% (219/231)
	*csgD*	96.5% (223/231)
Protein	*ompR*	100% (231/231)
	*bapA*	100% (231/231)
Quorum sensing	*pfs*	90.5% (209/231)
	*luxS*	100% (231/231)
Sigma factor	*rpoS*	100% (231/231)

### 3.3. Emergence of extensive MDR in SP isolates: alarming findings

The findings from the AST have revealed a worrisome pattern of extensive MDR among the SP strains. Figure 2C, illustrates the temporal surveillance of MDR in SP-BF from 2018 to 2022, indicating a distinct upward trend in MDR over this period, with an increasing prevalence of strains resistant to six, seven, or eight categories of antibiotics. Additionally, Figure 2D, presents a comprehensive analysis of the MDR phenotype in SP-BF strains. Notably, 100% (231/231) of the isolates exhibited resistance to at least one antibiotic, with an overwhelming majority (98.3%, 227/231) exhibiting MDR, and 84.4% (195/231) demonstrated resistance to four or more antibiotic categories. Alarmingly, a minute fraction (1.7%, 4/231) demonstrated an exceptionally severe level of resistance, rendering them resistant to all eight categories of antibiotics. The analysis of AMR rates for the tested antibiotics showed that the resistance rates to SIZ, SXT, and AMC exceeded 90%, while the strains displayed relatively high sensitivity to CRO, CIP, CTX, and NIT, with resistance rates lower than 20%. The results are summarized in Table 3.

**Table 3:** AST results of BF formation-positive SP strains for 17 antibiotics.

**Antibiotics**		**Breakpoints (μg/ml)**	**Results (%**, ***n*** = **231)**		
		**Intermediary**	**Resistance**	**Intermediary**	**Sensitive**
β-Lactams	AMP	14–16	87.4	0.9	11.7
	AMC	14–17	91.3	0.9	7.8
	CTX	15–22	16.0	14.7	69.3
	CAZ	15–17	19.9	3.0	77.1
	CRO	15–17	16.9	3.5	79.7
Sulfonamides	SXT	13–16	95.2	0	4.8
	TMP	11–15	85.7	3.0	11.3
	SIZ	13–16	96.1	1.3	2.6
Quinolone and fluoroquinolone	NAL	14–18	87.0	1.3	11.7
	CIP	16–20	16.5	9.5	74.0
Aminoglycosides	AKN	15–16	20.3	1.3	78.4
	STP	12–14	71.0	12.6	16.5
	KAN	14–17	28.1	6.5	65.4
	GEN	13–14	19.5	1.7	78.8
Nitrofuran	NIT	15–16	13.0	4.3	82.7
Tetracycline	TET	12–14	34.2	4.3	61.5
Chloramphenicol	FFC	13–17	43.7	3.5	52.8

### 3.4. Impact of BF formation on AMR: MIC and MBEC measurements

The results obtained from the MIC and MBEC measurements provide compelling evidence for the substantial increase in AMR following the formation of BF (Table 4). The comparison between the BF-forming strains and the planktonic bacteria revealed a remarkable 2- to 16-fold rise in the antibiotic concentration required to inhibit bacterial growth for the five antibiotics tested. After BF formation, it was observed that strong and moderate BF-forming strains exhibited a greater increase in resistance compared with weak BF-forming strains, indicating an enhanced resistance to CIP and GEN.

**Table 4:** Fold increase in inhibitory concentration after BF formation in nine SP isolates with different BF-formation capacities.

**Biofilm formation capacity**	**Number of strains**	**The difference in inhibitory concentration (fold)**				
		**CAZ**	**CIP**	**GEN**	**NIT**	**TET**
Strong biofilm producers	S1	-	4	4	4	-
	S2	-	2	2	-	2
	S3	-	2	2	-	2
Moderate biofilm producers	M1	-	16	4	2	4
	M2	2	16	2	-	-
	M3	2	2	-	4	-
Weak biofilm producers	W1	-	2	-	-	-
	W2	-	-	2	-	2
	W3	-	2	-	-	-

“-” indicates that there is no difference in the inhibitory concentration of this strain in the BF state and the planktonic state.

### 3.5. The OBFC and nutritional factors influencing BF formation

The experimental results showed the maximum BF formation at 37°C after 48 h of incubation, with an initial bacterial concentration of 1.2 × 10^6^ CFU/ml. The specific results are presented in Figure 2E. Under these optimal conditions, further investigations were then conducted to explore the influence of nutritional substances on BF formation. The findings revealed a gradual decrease in BF quantity as the mass fraction of NaCl increased. Moreover, the amount of BF formation exhibited a positive correlation with the increasing concentrations of glucose and TSB, without any observed decreasing trend.

## 4. Discussion

SP is a significant pathogen responsible for poultry diseases, posing substantial challenges to the poultry industry. Approximately 80% of chronic infections are associated with BF formation (22). On poultry farms, the BF facilitates the spread of SP and establishes a contamination cycle, with areas of high SP exposure, such as incubators and chick processing rooms in the hatchery, being particularly vulnerable (23). Feathers, dust particles, and egg trays and containers create optimal conditions for bacterial attachment and subsequent BF formation, thereby establishing persistent reservoirs of infection (24). This study indicates that a substantial number of SP strains possess the capacity to form BFs (36.8%, 231/627). Furthermore, the detection of moderate and strong BF-forming strains has exhibited a progressive increase in 2020–2022. However, in the investigation conducted by Guo *et al*., the results revealed that from 2011 to 2016, 53.3% of SP isolates (16/30) demonstrated weak BF producers, while none were classified as strong BF producers during 2011–2016 (25). The enhanced BF-forming capacity of SP strains over time may be attributed to an overall increase in the population's tolerance to adverse environmental factors, leading to the upregulation of BF-related genes. The BF-forming capacity of strains suggests their strong synthetic and metabolic capabilities; simultaneously, virulence proteins and factors associated with BF-formation are also upregulated, potentially contributing to higher pathogenicity of the strains (26). Further investigations are required to elucidate the specific factors contributing to these observations.

The process of BF formation is highly regulated and influenced by various signaling molecules and environmental factors. During adhesion and aggregation, key genes, such as proteins, curli fibers, and sigma factors, are upregulated, playing essential roles in the BF formation process. In our study, we found a significant positive correlation between the BF-related genes of strains and their BF formation capacity. Previous studies by Lu et al. (27) and Aleksandrowicz et al. (28) confirmed that strains knockout the *ompR* gene show a complete loss of BF formation capacity by inhibiting fimbriae and cellulose. Furthermore, sigma factors (*rpoS* gene) play a crucial role in regulating and integrating different environmental signals, ultimately affecting the synthesis rate of key proteins during BF formation (29). In our study, the detection rate of all these genes was 100% (231/231), suggesting their strong universality and potential importance in BF formation. The QS system is classified into AI-1, AI-2, and AI-3 based on different signaling molecules. Among them, AI-2 is considered the predominant universal signal for communication among Gram-negative bacteria (30). It is regulated by the *luxS* gene and the *pfs* gene, which encode the main catalytic enzyme responsible for AI-2 synthesis. In our investigation, the detection rate of the *luxS* gene was 100% (231/231), while the detection rate of the *pfs* gene was only 90.5% (209/231), suggesting that some strains may utilize alternative QS systems for BF formation (31). The observed differences in BF-forming capacity among different strains may be attributed to variations in gene expression or upregulation of other relevant genes. In studies focused on inhibiting BF formation, plant extracts effectively suppressed BF-related genes. Xu *et al*. demonstrated that berberine reduces BF formation by inhibiting the *fimA* gene expression. Moreover, quercetin also effectively inhibits BF formation by downregulating the expression of the *luxS* gene (32, 33). The highly detected genes in our study can be considered potential research targets for inhibiting SP-BF.

As a protective barrier, BF can activate adaptive stress responses in bacterial communities upon exposure to antibiotics, leading to rapid upregulation of resistance genes and a significant increase in AMR, contributing to the sustained presence of infections (34). Our study revealed a considerably high rate of MDR among SP strains in the wild (98.3%, 227/231). Additionally, observations after BF formation showed an increase in AMR ranging from 2-fold to 16-fold for some tested antibiotics. In comparison to the research findings by Gong et al. (35) and Penha Filho et al. (36), the severity of MDR and BF formation capacity of SP has been progressively increasing over the years. This trend poses a concerning issue as it limits treatment options and exacerbates the global challenge of AMR. The emergence of ARGs originating from farms also has the potential to transmit zoonotic pathogens, posing a direct threat to human health. Alarming statistics indicate that more than seven million people worldwide succumb to AMR-related complications each year (37). If left unchecked, the number of deaths may even surpass the mortality rate attributed to cancer in future.

The formation of BF is a complex process influenced by various nutritional factors and plays a crucial role in determining the speed and quantity of BF formation. The temperature has a significant impact on BF formation, and our research findings are consistent with those of Borges et al. (38) indicating that 37°C is the optimal temperature for BF formation. Moreover, our observation suggests that a high concentration of NaCl creates a high osmotic pressure environment, thereby impeding BF formation through its impact on bacterial vitality, which aligns with the study conducted by Iliadis et al. (39). Polysaccharides, as the primary constituents of EPS, are profoundly influenced by the availability of sugar sources. Our study further supports a positive correlation between the concentration of TSB and glucose and the quantity of SP-BF. These findings contribute to our understanding of the critical role that nutritional factors play in BF formation and offer practical implications for the development of targeted strategies to prevent and control bacterial BFs.

Our study highlights the urgent need for effective antimicrobial stewardship and infection control measures to address the underlying causes of MDR-SP and its rapid dissemination, particularly in poultry farms, which serve as a critical reservoir. In clinical practice, in addition to eradicating SP from the breeder flocks, it is crucial to develop targeted and reliable biosecurity measures aimed at inhibiting BF formation. To this end, the implementation of systematic and regular cleaning plans covering the entire production chain from the farm environment to the hatchery is paramount. Additionally, meticulous attention should be given to the thorough cleaning and disinfection of feed troughs and containers, as they can potentially serve as breeding grounds for pathogenic bacteria. Furthermore, by strategically combining physical interventions and the use of high-concentration NaCl, metal ions, and proteolytic enzymes as disinfectant additives, effective control of SP-BF formation and reduction of its spread can be achieved (40, 41).

## 5. Conclusion

In summary, this study conducted a comprehensive analysis of 627 SP strains isolated from the six large-scale chicken farms in Guangxi, China during 2018–2022. Among them, 36.8% (231/627) strains demonstrated the capacity to form BFs and exhibited a high level of MDR. These SP-BF strains were found to carry a high abundance of BF-related genes. Additionally, the investigation of OBFC revealed that 37°C is the most favorable temperature for SP-BF formation, and there is a positive correlation between glucose and TSB concentrations and the quantity of SP-BF. Conversely, NaCl exhibited an inverse relationship with BF formation. These findings underscore the importance of implementing effective preventive and control strategies to address SP-BF formation and MDR in the field. Moving forward, further research should focus on uncovering the regulatory mechanisms governing BF formation, investigating underlying pathways and key proteins, and conducting intervention studies targeting BF formation.

## Data availability statement

The original contributions presented in the study are included in the article/supplementary material, further inquiries can be directed to the corresponding author.

## Ethics statement

Ethical approval was not required for the study involving animals in accordance with the local legislation and institutional requirements because Strains used in this study were obtained from commercial chickens. The American Veterinary Medical Association did not require the study to be reviewed by an Ethics Committee because the experimental samples were obtained as a by-product of the poultry industry practices.

## Author contributions

WC performed the experiments and wrote the manuscript. ZX participated in the experimental design and interpreted the data. CL, CW, MW, and JL performed the partial experiment. PW contributed new reagents, analytic tools, and revised the manuscript. All authors read and approved the manuscript.
